# Friedreich’s Ataxia: A Neuronal Point of View on the Oxidative Stress Hypothesis

**DOI:** 10.3390/antiox3030592

**Published:** 2014-09-10

**Authors:** Barbara Carletti, Fiorella Piemonte

**Affiliations:** Unit of Neuromuscular and Neurodegenerative diseases, Children’s Hospital and Research Institute “Bambino Gesù”, 00165 Rome, Italy

**Keywords:** Friedreich’s ataxia, oxidative stress, neurodegeneration, antioxidants

## Abstract

A prominent feature of Friedreich’s ataxia (FRDA) is the neurodegeneration of the central and peripheral nervous systems, but little information is available about the mechanisms leading to neuronal damage in this pathology. Currently, no treatments delay, prevent, or reverse the inexorable decline that occurs in this condition. Evidence of oxidative damage has been demonstrated in Friedreich’s ataxia, and this damage has been proposed as the origin of the disease. Nevertheless, the role of oxidative stress in FRDA remains debatable. The lack of direct evidence of reactive oxygen species overproduction in FRDA cells and tissues and the failure of exogenous antioxidants to rescue FRDA phenotypes questions the role of oxidative stress in this pathology. For example, the antioxidant “idebenone” ameliorates cardiomyopathy in FRDA patients, but this therapy does not improve neurodegeneration. To date, no known pharmacological treatment with antioxidant properties cures or delays FRDA neuropathology. This review reports and discusses the evidence of oxidative stress in FRDA and focuses on the existing knowledge of the apparent ineffectiveness of antioxidants for the treatment of neuronal damage.

## 1. Introduction

Friedreich’s ataxia (FRDA) is an inherited autosomal recessive disorder caused by severely reduced levels of the mitochondrial iron chaperone protein, frataxin, as a result of a large GAA triplet-repeat expansion within the first intron of the frataxin gene. The GAA expansion interferes with gene transcription and determine a reduction in the amount of frataxin protein. The residual amount of frataxin protein in FRDA patients varies between 5% and 35% of normal levels. The prominent feature of FRDA pathology is neurodegeneration in the central and peripheral nervous systems. A distal length-related axonal degeneration affects the upper motor neurons of the corticospinal tract, the posterior columns of the spinal cord, the spinocerebellar tract and the large sensory fibers of the peripheral nerves [1]. Clinical symptoms appear during childhood or adolescence, and they are characterized by a progressive relentless ataxia associated with dysarthria, loss of reflexes and decrease of deep sensation [2]. Patients typically require wheelchairs within 10 to 20 years after symptoms begin. A hypertrophic cardiomyopathy is the second important clinical trait that presents in two-thirds of the patients, and it is the most frequent cause of early death. Additional non-neurological features are skeletal deformities and diabetes mellitus. The similarities of FRDA with vitamin E deficiency suggested from the beginning a role of oxidative damage in the pathogenesis of this disorder [3]. However, a prolonged debate began to emerge as the importance of oxidative stress in FRDA became increasingly evident. Is oxidative stress a secondary event of disease progression, or does it represent the primary cause of cell damage in the target tissues? If oxidative damage contributes to the pathogenesis of neurodegeneration in FRDA, why are antioxidant therapies unsuccessful in ameliorating nervous system clinical symptoms? This review provides an overview of the oxidative stress findings in FRDA and analyzes the possible role of redox markers in FRDA neuronal damage to delineate the cause of the selective vulnerability of specific neurons to frataxin deficiency and their apparent unresponsiveness to antioxidants.

## 2. Frataxin Function

Oxidative stress is caused by an imbalance between the production of reactive oxygen species (ROS) and the cellular antioxidant defense, which usually detoxifies reactive intermediates and repairs the resulting damage. ROS production occurs during normal cellular aerobic metabolism, primarily during mitochondrial respiration during the incomplete reduction of oxygen to water. ROS includes both free radicals that contain a highly reactive unpaired electron, such as superoxide anion (O_2_^−^) and hydroxyl radical (OH), and other molecular species, such as hydrogen peroxide (H_2_O_2_). All of these species react with cellular components that oxidize proteins, lipids and DNA [4]. The cell controls the flux of ROS under normal physiological conditions by neutralizing and preventing ROS production to maintain the redox equilibrium. Oxidative stress occurs when ROS levels overwhelm the antioxidant systems. A vicious circle was hypothesized very early on that considered oxidative stress as primarily responsible for the cellular consequences of frataxin loss of function [5,6].

Frataxin is a ubiquitous mitochondrial protein involved in intracellular iron homeostasis that participates in heme biogenesis [7], iron binding storage and detoxification [8,9], iron chaperone activity [10], and the biosynthesis of iron-sulfur complexes (ISCs) [11,12,13,14,15,16,17,18]. The proposed interpretation for FRDA pathogenesis focuses on ISC biosynthesis. ISCs act as prosthetic groups for several proteins that participate in energy metabolism, respiration, iron metabolism, purine synthesis and DNA repair. Frataxin likely contributes to ISC assembly by making ferrous iron available [10]. Several studies suggest that a frataxin deficiency underlies an inefficient use of iron during ISC synthesis with a consequent accumulation of the metal and defects in ISC-containing enzymes [19,20]. Frataxin involvement in Fe/S formation was confirmed by ISC enzyme impairment in FRDA patients’ tissues and yeasts and animal models [5,21,22,23]. These studies showed particular dysfunction in Fe/S containing enzymes such as aconitase and complexes I, II and III of the mitochondrial respiratory chain with consequent defects in mitochondrial respiration, lower ATP production and iron accumulation.

The observations of mitochondrial enzyme deficiencies and iron accumulation in animal models and affected tissues suggested that the loss of frataxin determined the presence of unbound reactive free iron, which generates ROS within the mitochondrion via the Fenton reaction [23,24]. This process would lead to oxidative damage which might inactivate mitochondrial enzymes and determine a further generation of free radicals. In fact, Fe/S enzymes of the mitochondrial respiratory chain comprise the major ROS generators and are thus more exposed to the oxidative damage. ROS production during mitochondrial respiration consists of prematurely leaking electrons, mostly at complexes I and III. Electrons react with molecular oxygen to generate the superoxide anion (O_2_^−^). Frataxin deficiency in FRDA underlies the dysfunctional biogenesis of the ISC-containing complexes with reduced activity of the mitochondrial respiratory chain enzymes. This process presumably enhances superoxide generation, which can be converted to H_2_O_2 _by the dismutation catalyzed by superoxide dismutase (SOD) or Fe^3+^. The excess of Fe^2+^ and Fe^3+^ derived from the frataxin defect may generate more toxic OH from the hydrogen peroxide via the Fenton reaction (Fe^2+^ + H_2_O_2_→ Fe^3+^ + OH + OH^−^ or Fe^3+^ + H_2_O_2_→ Fe^2+^ + OOH + H^+^), which causes further respiratory chain damage and ROS production. Several aspects of frataxin function remain controversial, but it is now commonly accepted that frataxin prevents the highly redox-reactive iron from oxidative stress generation, thus frataxin deficiency might render the cell more vulnerable to oxidative stress [16].

## 3. Evidence for Oxidative Stress in FRDA

Unequivocal findings for increased ROS production in frataxin-deficient cells are lacking. However, several markers of oxidative stress have been revealed in FRDA cells and patient tissues. Increased plasma level of malondialdehyde (a lipid peroxidation product) [25] and increased urinary 8-hydroxy-2′-deoxiguanosine (a marker of oxidative DNA damage) were found in FRDA patients [26]. Additional confirmations also came from the dysregulation of glutathione homeostasis. Glutathione is a critical component of antioxidant cellular defense, and one key aspect of the redox status is the equilibrium between the reduced and oxidized forms of this tri-peptide (GSH/GSSG). The increase in the oxidized form of GSSG during oxidative stress conditions induces a process termed glutathionylation, which forms mixed disulfides between glutathione and protein cysteine residues. Our studies using blood or cells from FRDA patients suggest that frataxin deficiency leads to an impairment of glutathione equilibrium [27,28]. A considerable reduction in free glutathione levels and a significant increase in glutathione bound to hemoglobin were observed in the blood of FRDA patients, and GSSG concentrations increase in cultured fibroblasts and lymphoblasts from FRDA patients. We also associated the loss of cytoskeletal organization to the constitutive glutathionylation of cytoskeletal proteins and its increase in FRDA fibroblasts and spinal cord compared to healthy controls [29,30,31]. The altered glutathione status was further confirmed by analyses of yeast and human frataxin-deficient cells showing a low GSH/GSSG ratio, thiol modification of key mitochondrial proteins and changes in GSH-dependent enzymes [32,33].

A direct demonstration of increased ROS production in FRDA is limited to two studies that showed an overproduction of peroxide in patients’ lymphoblasts and frataxin-deficient P19 cells [34,35]. Although we have scarce information on ROS production in FRDA, a wide variety of frataxin-deficient cells and FRDA tissues show an oversensitivity to oxidants, which indicates that they are under oxidative stress conditions [36,37,38,39]. Anderson and collaborators [40] reported that the administration of H_2_O_2_, but not superoxide, scavenging enzymes rescue the pathological phenotype in a drosophila FRDA model, which suggests hydrogen peroxide as the reactive species involved in FRDA damage.

In addition to the possibility of ROS overproduction, several reports also highlighted a disabled recruitment of antioxidant defenses. An impaired induction of SOD under oxidative stimuli has been reported in FRDA fibroblasts [37,41], and a significant increase in the activity ratio between SOD and glutathione peroxidase (GPX) has been detected by our group in the blood of FRDA patients [42]. SOD plays a fundamental role in modulating ROS toxicity as catalyzes the dismutation of superoxide into oxygen and hydrogen peroxide, subsequently reduced to water by GPX. Thus, a disequilibrium in SOD activity might alter the redox status of the cell towards oxidative stress. A further demonstration of impaired antioxidant defense in FRDA cells was provided also by Paupe and colleagues (2009) which reported that FRDA fibroblasts showed disorganization and impairment of the Nrf2 signaling pathway [43]. Nrf2 is a key transcription factor that responds to oxidants by binding to the antioxidant responsive element (ARE) and activating the transcription of Phase II antioxidant genes. These genes encode for several enzymes, such as SOD, catalase and the enzymes responsible for glutathione synthesis. Nrf2 does not translocate to the nucleus in FRDA fibroblasts or induce the transcription of antioxidant enzymes under oxidative stress conditions, which increases their vulnerability. Similarly, Marmolino *et al.* (2010) showed that frataxin-deficient cells presented reduced levels of PGC-1α, a transcription factor that emerged as a key player in the induction of several antioxidant pathways [44]. PGC-1α is a transcriptional regulator of mitochondrial biogenesis and metabolism that ensures a global positive impact on oxidative metabolism by increasing mitochondrial functions and minimizing the buildup of its oxidant by-products [45]. This specific pathway represents a physiological response activated by ROS that might be impaired in FRDA, where a decrease in PGC-1α expression is suggested to result in oxidative sufferance for diminished ROS-detoxifying capacity. These observations support the possibility of a further ROS overload due to an inadequate contribution of the antioxidant defense system. Therefore, whether oxidative status in FRDA arises from a ROS overproduction or a disabled antioxidant defense or both, an increasing body of evidence indicates that this overload may underlie the cell damage and play a key role in the pathogenesis of this disorder.

## 4. Neuronal Damage

Antioxidant therapy received increasing attention in clinical neurology, and many trials have been performed in common neurologic disorders as well as rare conditions supposed to be associated to oxidative stress [46]. However the lack of a proper understanding of the complex free radical biochemistry in neuron diseases determined conflicting and unsuccessful results. Unfortunately, this condition is also common to Friedreich’s ataxia.

The successful results of the treatment of FRDA patients with the antioxidant idebenone were initially very encouraging. An early trial demonstrated that low doses of idebenone reduced cardiac hypertrophy in the majority of patients [26,45,47,48,49], but no influence was detected on the neurological progression. A comprehensive review of all randomized clinical trials using antioxidant drugs confirmed that none of the tested pharmacological treatments had significant neurological benefits in FRDA patients [50]. This disappointing translation of the oxidative stress hypothesis into useful antioxidant therapy to ameliorate the neurological symptoms suggests more complex cellular interactions than initially thought.

FRDA neuropathology includes atrophy of the dorsal root ganglia (DRG) with a progressive destruction of the larger neurons and thicker myelinated axons, which accounts for the thinning of the dorsal root and sensory nerve neuropathy [51,52]. The impact of DRG degeneration on the downstream structures of the spinal cord and brainstem underlies secondary damage, with atrophy of the Clarke and dorsal column, gracile and cuneate nucleus and spinocerebellar tract. Atrophy of Betz cells and the corticospinal tract is the second purely intrinsic CNS lesion of FRDA. How does a global event, such as oxidative stress, result in the selective loss of specific neurons? In addition, FRDA is a genetic disorder, and frataxin expression in different tissues does not explain this selective vulnerability. We might speculate that DRG neurons and upper motor neurons are among the longest in the body, and therefore, present a high-energy demand for axonal transport. A high ATP requirement combined with mitochondrial dysfunction may render these neurons more vulnerable to oxidative stress and degeneration than other neuronal groups. This hypothesis is consistent with the neurodegenerative phenotype associated with a selective defect of the retrograde axonal transport reported in a drosophila model of FRDA [53]. These authors tracked individual organelles along the axon using mito-GFP and showed that retrogradely moving mitochondria were profoundly affected with an excess accumulation at the neuromuscular junctions. Shidara and colleagues [53] suggested that the transport deficiency is an effect of a deficient mitochondrial metabolism and an inadequate production of ATP. In fact, mitochondria accumulation in the distal axons and synapses are profoundly depolarized and denies an adequate energy support.

Nevertheless, scarce data are available on the mechanisms of neuronal damage in FRDA pathology, and clear indications of oxidative damage in neurons, which paralleled existing data on other FRDA cell types, were obtained with difficulties. In 2000, Bradley and collaborators [54] performed a comprehensive analysis of mitochondrial respiratory chain and aconitase activities from FRDA frozen tissues, including cerebellum and DRG, but surprisingly, they detected normal activity values in DRG samples. These results were most likely justifiable by the loss of the larger ganglion cells at the time of sample collection. To date, dysfunctional mitochondrial respiratory chain activities have not been detected in FRDA target neurons, and the analysis of iron overload did not evidence a clear accumulation of this metal in neurons. However, an altered distribution of Fe-handling proteins has been found, which suggests a metal transfer from degenerating DRG neurons to activated satellite cells [55]. Nonetheless, an accurate analysis performed on DRG cells from the YG8R mouse model evidenced oxidative stress caused by impaired glutathione redox status and antioxidant protection [56]. Microarray analysis of DRG tissue identified decreased transcripts encoding several antioxidant enzymes and a significant reduction in Nrf2 expression. Consistent with these data, we also found a defective Nrf2 expression in frataxin-deficient NSC34 neuronal cells after treatment with oxidized glutathione and a reduced basal expression of this transcription factor compared to control cells [57]. In addition, by analyzing the patterns of glutathionylation in the spinal cords of FRDA patients, Sparaco *et al.* (2009) found that cytoskeletal abnormalities co-localized with an increase in protein glutathionylation, which suggested a role for glutathione in FRDA pathogenesis [30].These results confirm the presence of oxidative stress in FRDA neurons and encourage the investigation of the source of ROS generation in the target cells of nervous tissue. These results also highlight that oxidative stress is a global event that is likely to produce many different downstream effects collectively and depict a complex neurodegenerative scenario.

The consequences of oxidative stress may be different and numerous, but several studies evidenced a common final outcome of the neurodegenerative cascade in the apoptotic pathways that most likely occurs in FRDA neuropathy. An increased susceptibility to apoptosis has been detected in fibroblasts and lymphoblasts from FRDA patients and frataxin-deficient cell lines [35,36,58,59,60], but a more stringent result came from two recent works on neuronal cells. Palomo *et al.* (2011) knocked down frataxin expression in the human neuroblastoma cell line SH-SY5Y using lentiviral vectors [61]. The SH-SY5Y cell line retain the ability to stop proliferating under specific condition, and to differentiate into postmitotic cells with the characteristic features of mature neurons. These authors demonstrated that differentiated neuron-like cells were very susceptible to oxidative stress and underwent a characteristic apoptotic cell death accompanied by p53 induction and caspase 3 activation. The authors argue that oxidative stress may play a causative role in p53 activation, which triggers the apoptotic cascade through the expression of the downstream genes PUMA and Bax. This work extended a previous finding of an enhanced apoptosis in frataxin-deficient embryonic P19 stem-like cells induced to differentiate into neurons [35]. More recently, Mincheva-Tasheva and collaborators explored the neurodegenerative process occurring in primary rat DRG neurons after frataxin depletion and observed an apoptotic cell death associated with a marked increase in intracellular Ca^2+^ levels (2013) [62]. The authors did not investigate the possible involvement of oxidative stress, but it has long been known that ROS can alter Ca^2+^ homeostasis and cause an increase in the intracellular ion concentration, which may disrupt normal metabolism and lead to cell death.

The notion that ROS may act on redox-sensitive transcription factors to alter the pattern of gene expression and determine the decision between cell survival and death increases the complexity of the dangerous mechanisms potentially activated by oxidative stress and introduces several further considerations for successful antioxidant therapies. The apoptotic pathway represents the last stage of the FRDA neuronal response to a chronic oxidative stress condition and a downstream target in the neurodegenerative cascade. The neurodegenerative process is the result of a progressive attrition of dysfunctional neurons that, although triggered from an oxidative insult, may then progress and evolve over time in an oxidative-independent manner. This pathway implies that the search for early events in oxidative stress-mediated pathogenesis is the real focus for the development of beneficial antioxidant therapies. Antioxidants should directly block the origin of the oxidative damage and be administered at an early stage before the neurodegenerative process begins. A clear understanding of the mechanisms generating oxidative stress in FRDA target neurons is essential for an efficacious antioxidant therapy.

Oxidative stress is most likely differentially generated in diverse tissues and directly or indirectly initiates several dangerous cell type-specific metabolic pathways, which may frustrate the general use of antioxidants not specifically targeting the cause of the damage. One possible explanation for the unsuccessful treatment of the neurological symptoms with idebenone may reside in a similar phenomenon. Idebenone is a short-chain coenzyme Q that acts as a substrate for complexes II and III and ameliorates the function of these respiratory chain proteins. In 1997, Rustin *et al.* demonstrated that idebenone protected complex II, lipids and aconitase from iron injury in heart homogenates [5] and, encouraged by these findings, the authors observed a significant reduction of hypertrophic cardiomyopathy in three treated patients [47]. Complexes II, III and aconitase activities were impaired in FRDA heart tissue, and the beneficial effects of idebenone prove the success of a treatment directly targeting one of the possible causes of damage in cardiac cells. Nevertheless, the pathogenesis may be not the same in the nervous tissue. For example, idebenone marginally inhibits complex I and strongly favors succinate oxidation [63]. We recently found that mitochondrial complex I is severely affected in frataxin deficient NSC34 motoneuronal cells [64]. In our model the NSC34 cell line has been silenced for frataxin gene by means of specific shRNA lentiviral vectors and reproduced some major biochemical and morphological features of FRDA. Whether this model recapitulate the mitochondrial damage in FRDA target neurons, idebenone treatment by inhibiting complex I may worsen the condition of the nervous tissue at the target sites. Therefore, a systematic analysis of potential sites and mechanisms of increased ROS production and impaired cellular antioxidant defense systems in FRDA damaged neurons is demanded. A limit to the effectiveness of antioxidants may be for example that they randomly diffuse through the cell, with only a small proportion of them reaching the site of damage. Accordingly, Jauslin and colleagues (2003) demonstrated that targeting antioxidants to mitochondria improve their effectiveness [65]. Thus, a subcellular compartmentalization of oxidative sites is critical to direct effective therapeutic treatments, and an analysis of *in vivo* imaging of redox-sensitive proteins targeted to the cytosol, mitochondrial matrix and the intermembrane space should be performed. These investigations may enable the dissection of the pathogenetic mechanisms and the role of oxidative stress in FRDA neuron damage and enable the identification of possible neuron-specific antioxidant molecules.

## 5. Conclusions

Despite the mounting evidence that oxidative stress is involved in the FRDA neuron damage that contributes to the pathogenesis of this disorder, clear research documenting sites and mechanisms of oxidative stress generation is lacking. Recent reports highlighted significant reductions in antioxidant protection and impairments in the glutathione redox status in frataxin-deficient neurons, but a causative mechanism documenting the role of oxidative stress in determining the FRDA neurodegenerative cascade is far from being discovered. The new findings on the apoptotic pathways as the final result of FRDA neuron damage definitely opens a complex scenario and suggests that oxidative stress most likely triggers many different downstream effects ending in a common outcome. Therefore, the early events in the oxidative process could prove decisive for an efficacious therapy targeting oxidative stress, whereas the final stages of neurodegeneration may not be responsive to an antioxidant treatment. For this reason, we remark that major efforts should be performed to elucidate the mechanisms of ROS production in FRDA. An efficacious antioxidant therapy should act upstream of the neurodegenerative process at the origin of oxidative stress generation to interrupt the dangerous cellular interactions of ROS and prevent the breakdown of target enzymes that trigger the neurodegenerative pathways. A general use of antioxidants that act as scavengers rather than blockers of the source of ROS generation may be ineffective. A more complete knowledge of the pathogenetic mechanisms induced by oxidative stress and the causes underlying these processes are needed to enable the development of useful antioxidant treatments. Only then we can identify molecules with suitable pharmacological antioxidant properties.
